# Neutrophil‐to‐lymphocyte ratio and longitudinal cognitive performance in Parkinson's disease

**DOI:** 10.1002/acn3.52144

**Published:** 2024-07-19

**Authors:** Jenniffer Lucero, Ashita Gurnani, Janice Weinberg, Ludy C Shih

**Affiliations:** ^1^ Department of Neurology Boston University Chobanian and Avedisian School of Medicine Boston Massachusetts 02118 USA; ^2^ Department of Neurology Boston Medical Center Boston Massachusetts 02118 USA; ^3^ Department of Biostatistics Boston University School of Public Health Boston 02118 Massachusetts USA

## Abstract

**Objective:**

Previous studies have suggested a link between peripheral inflammation and cognitive outcomes in the general population and individuals with Parkinson's disease (PD). We sought to test the association between peripheral inflammation, measured by the neutrophil‐to‐lymphocyte ratio (NLR), cognitive performance, and mild cognitive impairment (MCI) status in individuals with PD.

**Methods:**

A retrospective, longitudinal analysis was carried out using data from the Parkinson's Progression Markers Initiative (PPMI), including 422 participants with PD followed over 5 years. Cognitive performance was assessed using a neuropsychological battery including the Montreal Cognitive Assessment (MoCA) and tests of verbal learning, visuospatial function, processing speed, and executive function. Mixed‐effect regression models were used to analyze the association between NLR, cognitive performance, and MCI status, controlling for age, sex, education, APOE genotype, and motor severity.

**Results:**

There was a negative association between NLR and MoCA, even after adjusting for covariates (*b* = −0.12, *p* = 0.033). MoCA scores for individuals in the high NLR category exhibited a more rapid decline over time compared to the low NLR group (*b* = −0.16, *p* = 0.012). Increased NLR was associated with decreased performance across all cognitive domains. However, NLR was not associated with MCI status over 5 years of follow‐up.

**Interpretation:**

This study demonstrates a link between elevated NLR and cognitive performance in PD, but not with MCI status over 5 years. This suggests that NLR is more strongly associated with day‐to‐day cognitive performance than with incident MCI, but this requires further study in more heterogeneous cohorts.

## Introduction

Although Parkinson's disease (PD) is a progressive neurodegenerative disorder best recognized by motor symptoms, such as tremor, rigidity, and bradykinesia, non‐motor symptoms including cognitive impairment are common.^1^ Mild cognitive impairment (MCI) affects around 20–40% of individuals diagnosed with PD depending upon its definition, with an annual rate of conversion to dementia ranging from 8% to 16%.^2^, ^3^, ^4^, ^5^ Cognitive impairment manifests on a spectrum,^6^ ranging from mild deficits in cognitive performance – on tests assessing global cognition or specific cognitive domains, such as visuospatial function, attention, and executive function – to dementia. Both global and domain‐specific cognitive deficits have the potential to significantly impact independence in activities of daily living.^7^

PD‐MCI, as defined by the Movement Disorder Society (MDS) Task Force diagnostic criteria through use of either a brief or comprehensive neuropsychological battery,^8^ is common, even at time of PD diagnosis. A recent comprehensive meta‐analysis reported a prevalence of 40% of PD‐MCI using these criteria across 41 studies comprising 7053 individuals diagnosed with PD.^9^ Longitudinal studies, like the Norwegian ParkWest Study revealed the baseline prevalence of PD‐MCI to be 20% at the time of diagnosis^10^ with nearly 40% of these patients progressing to dementia at 5‐year follow‐up.^5^

Peripheral inflammation may be a risk factor for MCI and dementia in the general population,^11^, ^12^ where it has been associated with both incident and prevalent Alzheimer's disease (AD). Less clear is whether this holds true in PD, though some preliminary studies suggest this may be the case.^13^, ^14^ Careful consideration of other known risk factors for dementia in PD is required and should include age, male sex, low education level, visual hallucinations, REM sleep behavior disorder (RBD), prolonged disease duration, severe motor impairment, heart disease, and apolipoprotein epsilon 4 (APOE ε4) allele status, the most prominent genetic risk factor for sporadic AD.^15^, ^16^, ^17^, ^18^, ^19^, ^20^, ^21^, ^22^ Exactly which mechanisms peripheral inflammation influences PD disease course is still being explored.^23^, ^24^

Peripheral leukocytes may activate inflammatory cascades or directly infiltrate brain parenchyma.^24^ Neutrophils play a role as one of the primary participants in the innate immune response to pathogens,^25^ while lymphocytes are known to be responsible for adaptive immunity. The neutrophil‐to‐lymphocyte ratio (NLR) is easily obtained by standard laboratory peripheral blood lymphocyte count. NLR has been associated with prevalent and incident AD,^11^, ^26^, ^27^ as well as with PD in cross‐section, compared to healthy controls.^13^, ^28^, ^29^, ^30^ Additionally, higher NLR has been associated with PD with MCI compared to without, in a small cross‐sectional study.^13^ In the Parkinson's Progression Markers Initiative (PPMI) study following 423 individuals with de novo PD cohort over 2 years, decreased lymphocyte count was associated with decline in global cognitive performance over 2 years, but only in APOE e4 allele carriers, while NLR was not specifically examined.^13^, ^14^ Whether NLR is a predictor of global cognitive impairment or amnestic changes more typical of AD pathology versus non‐amnestic changes over time is not known.

We therefore sought to test the association between NLR and 5‐year trends in longitudinal global cognitive performance, domain‐specific cognitive performance, and MCI status. For this study, we used the PPMI, an ongoing extensive, observational, multicenter, longitudinal prospective study that includes de novo PD patients and healthy controls (HC)^6^ with extensive risk factor and blood biomarker data including blood counts.

## Methods

### Study design and population

This longitudinal, retrospective cohort study was carried out using data extracted from the PPMI database. The PPMI study, initiated in 2010, enrolled 423 new, untreated PD patients with the methods described extensively elsewhere.^31^ Eligible participants were 30 years or older, with a diagnosis of PD for 2 years or less at the screening visit, not requiring PD medication for at least 6 months, and exhibited at least two symptoms like resting tremor or bradykinesia, and Hoehn and Yahr stage I or II at baseline. Exclusion criteria included current or recent use of PD medications, a history of atypical parkinsonian syndromes, or a clinical diagnosis of dementia. Ethical approval was provided by the University of Rochester IRB.

**Figure 1:**
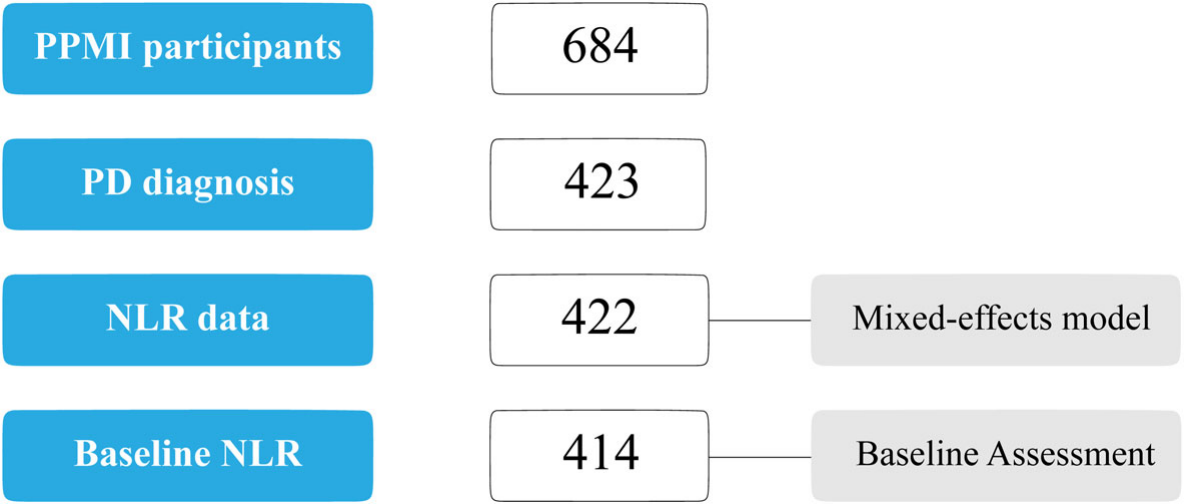
Study population. Participants with neutrophil‐to‐lymphocyte ratio (NLR) data at any point were included in the analysis using linear mixed‐effects models. Participants with NLR data at baseline were included in the baseline assessment.

For this study, we included data from 422 participants with PD who underwent semiannual visits spanning a 5‐year period (2011–2016), with comprehensive clinical information including demographics, laboratory values, clinical outcomes assessments, and an abbreviated neuropsychological battery including Montreal Cognitive Assessment (MoCA) and several other tests assessing various cognitive domains. There were 414 PD participants who had data on neutrophil and lymphocyte counts to calculate NLR at baseline. Participants had neutrophil and lymphocyte count data at subsequent visits and were included in the linear mixed‐effects models to analyze the relationship between the variables across multiple visits in a total of 422 participants (Fig. 1).

### Variables of interest

The primary outcome of this study is global cognitive performance as measured by the MoCA.^32^, ^33^ Based on earlier literature,^34^ MoCA score of 26 was employed as a threshold to identify cognitive impairment, with a sensitivity of 98% and a specificity of 52% for identifying cognitive impairment. However, we examined MCI status determined if a participant scored >1.5 SD below norm on two cognitive tests, meeting Level I criteria for MCI, as endorsed by the MDS Task Force on PD‐MCI.

We also used the Symbol Digit Modalities Test (SDMT) and Hopkins Verbal Learning Test (HVLT) Delayed Recall, Judgment of Line Orientation (JOLO), Letter Number Sequencing (LNS), and Semantic Fluency (SF) as secondary outcomes. The SDMT is a neuropsychological assessment tool that is often used to evaluate cognitive function, particularly information processing speed and working memory. The HVLT Revised‐Delayed Recall is a widely used neuropsychological assessment for evaluating verbal memory, particularly focusing on the ability to recall information after a delay. The HVLT Delayed Recall is sensitive to early cognitive changes, especially in verbal memory.^35^, ^36^, ^37^ The JOLO is a widely used test of visuospatial function that uses minimal motor response and has shown excellent test–retest reliability in PD.^38^ Letter Number Sequencing is test of working memory capacity and is a subtest of the Wechsler Adult Intelligence Scale (WAIS‐IV).^39^ SF is widely considered to be a test of executive function, involving verbal retrieval and self‐monitoring, and is significantly impaired in PD.^40^, ^41^

For the NLR, a cutoff value of 3 was used based on previous studies suggesting NLR >3 was indicative of peripheral inflammation in the general population.^42^, ^43^, ^44^ Factors such as age, sex, and underlying medical conditions can be associated with NLR. For the purposes of this analysis, NLR values were dichotomized as low NLR (≤3) and high NLR (>3). We incorporated additional covariates that have been previously associated with the risk of cognitive impairment and dementia, including age,^45^ sex,^46^ education,^45^ APOE genotype,^47^ RBD,^48^, ^49^ and PD motor severity as measured by Movement Disorders Society Unified PD Rating Scale (MDS‐UPDRS) Part III Motor Scale, assessed in the off‐medication state, as previous studies suggest associations between severity of motor symptoms and future cognitive decline.^50^

### Statistical analysis

For our analytical approach, descriptive statistics were used to depict the characteristics of the study cohort at baseline. For baseline differences between high and low NLR groups, we used a Welch's two sample *t*‐test and logistic regression depending on the variable type. All analyses were carried out using R Studio.

To account for data from multiple visits for each participant, we adopted mixed‐effects regression models. These models aimed to examine the association between MoCA scores and NLR longitudinally across the multiple visits during the 5‐year observation period. The model encompassed controlling for potential covariates, namely age, sex, years of education, MDS‐UPDRS, APOE e4 genotype, and RBD. We included covariates linked to both NLR and cognitive impairment in the model and added the duration of PD after baseline analysis showed differences between the high and low NLR groups. We used scatter plots and residual plots to assess the linearity of the relationship between NLR and MoCA scores. Longitudinal associations between NLR and MoCA was explored using a linear mixed‐effects models. A primary analysis was conducted without covariates. After the primary analysis, we increased the complexity of the mixed‐effects model to include the covariates. To avoid overfitting, we limited the number of variables by removing irrelevant predictors. In a second mixed‐effects model, we categorized NLR into groups (high and low) to facilitate interpretation and potential clinical applicability of the findings. To explore the variation in the effect of NLR on MoCA score over time, we added a time interaction (year) to the mixed‐effects model which allowed us to assess whether changes over time in MoCA scores differed between the high and low NLR groups. Other cognitive endpoints were evaluated employing similar methods. Initially, NLR was treated as a continuous variable, and subsequently, it was categorized into low and high NLR groups. Additionally, an interaction term (year) was incorporated into the model, mirroring the methodology applied to the primary outcome (MoCA).

We used a logistic regression mixed‐effects model with time interaction to examine the relationship between NLR levels and MCI status, as defined by MDS PD‐MCI Level 1 criteria (at least two cognitive test scores >1.5 standard deviations below standardized mean), excluding those with MCI status at baseline. Additionally, a linear mixed‐effects model examined the longitudinal association between NLR and our primary outcome, MoCA score, in this reduced sample.

## Results

Baseline characteristics and between‐group comparisons within the entire cohort are presented in Table 1. Several variables exhibited statistically significant differences (*p* < 0.05) between the low NLR and high NLR groups. The following variables were significantly associated with high NLR: older age at baseline (*p* = 0.029), male sex (*p* = 0.004), longer duration of PD from diagnosis to enrollment in the study (*p* = 0.005), older age at PD diagnosis (*p* = 0.045), and a higher MDS UPDRS Part III score (*p* = 0.012). At baseline, SDMT scores were significantly lower in the high NLR group, although there were no significant differences in the MoCA or the Hopkins Verbal Learning Test (HVLT) scores between both the groups (Table 2).

**Table 1:** Baseline characteristics of study participants.^a^

Variable	PD subjects *n* = 414	Low NLR *n* = 301	High NLR *n* = 113	*p*‐Value
Age				
Mean (SD)	61.72 (9.6)	61.11 (9.9)	63.36 (8.96)	0.029***
(Min, max)	(33.5, 84.9)	(33.5, 82.98)	(33.72, 84.88)	
Sex (no, %)				
Male	272 (65.7%)	185 (61.46%)	87 (76.99%)	0.004***
Female	142 (34.3%)	116 (38.54%)	26 (23.01%)	
Race (no, %)				
White	382 (92.27%)	274 (91.03%)	107 (94.7%)	0.608
Black	6 (1.45%)	5 (1.66%)	1 (0.88%)	
Asian	8 (1.93%)	6 (1.99%)	2 (1.77%)	
Other	18 (4.35%)	15 (4.98%)	3 (2.65%)	
Education years				
Mean (SD)	15.5 (2.9)	15.52 (3.11)	15.58 (2.41)	0.885
(Min, max)	(5, 26)	(5, 26)	(9, 23)	
Duration of PD from diagnosis to enrollment (months)				
Median (IQR)	4.18 (5.22)	3.73 (4.37)	5.13 (8.6)	0.005***
(Min, max)	(0.4, 35.83)	(0.4, 34.77)	(1.13, 35.8)	
Age at PD diagnosis				
Median (IQR)	62.10 (13.88)	61.67 (14.53)	63.07 (12.46)	0.045***
(Min, max)	(31, 84)	(33, 82)	(31.84, 84.79)	
Age at PD symptom onset				
Median (IQR)	60.56 (13.71)	60.28 (14.47)	63.07 (8.95)	0.067
(Min, max)	(25, 83)	(25, 81)	(30.99, 83.01)	
APOE genotype (no, %)				
e2/e2	2 (0.48%)	2 (0.66%)	0	0.594
e2/e4	7 (1.69%)	5 (1.66%)	2 (1.77%)	
e3/e2	51 (12.32%)	39 (12.96%)	12 (10.62%)	
e3/e3	223 (53.86%)	153 (50.83%)	70 (61.94%)	
e4/e3	83 (20.04%)	64 (21.26%)	19 (16.81%)	
e4/e4	9 (2.17%)	8 (2.66%)	1 (0.88%)	
MDS‐UPDRS total score				
Median (IQR)	31 (18)	30 (18)	33 (18)	0.007***
(Min, max)	(7, 72)	(7, 72)	(12, 70)	
MDS‐UPDRS Part I				
Median (IQR)	5 (4)	5 (4)	5 (5)	0.15
(Min, max)	(0, 24)	(0, 24)	(0, 23)	
MDS‐UPDRS Part II				
Median (IQR)	5 (5)	5 (5)	6 (6)	0.097
(Min, max)	(0, 22)	(0. 22)	(1, 20)	
MDS‐UPDRS Part III				
Median (IQR)	20 (12)	19 (12)	21 (12)	0.012***
(Min, max)	(4, 51)	(4, 46)	(7, 51)	
Hoehn and Yahr stage (no, %)				
Stage 1	181 (43.72%)	136 (45.18%)	45 (39.82%)	0.345
Stage 2	231 (55.8%)	163 (54.15%)	68 (60.18%)	
Stage 3–5	2 (0.48%)	2 (0.66%)	0	
RBD (frequency, %)				
RBD	153 (36.96%)	111 (26.81%)	42 (37.17%)	0.969
No RBD	258 (62.32%)	186 (44.93%)	71 (62.83%)	

APOE, apolipoprotein E; IQR, interquartile range; NLR, neutrophil‐to‐lymphocyte ratio; PD, Parkinson's disease; RBD, REM sleep behavior disorder; SD, standard deviation.

^a^ Data derived from the Parkinson Progression Marker Initiative (PPMI).^7^

*** *p*‐Value <0.05.

**Table 2:** Baseline participant cognitive performance scores.^a^

Variable	PD subjects *n* = 414	Low NLR *n* = 301	High NLR *n* = 113	*p*‐Value
MoCA score				
Median (IQR)	27 (3)	27 (3)	27 (3)	0.500
(Min, max)	(17, 30)	(17, 30)	(17, 30)	
Symbol Digit Modalities Test				
Mean (SD)	41.1 (9.6)	41.71 (9.71)	39.44 (9.58)	0.034 ***
(Min, max)	(7, 82)	(15, 82)	(7, 63)	
HVLT Immediate/Total Recall				
Mean (SD)	24.5 (4.9)	24.67 (5)	23.8 (4.84)	0.108
(Min, max)	(9, 36)	(9, 36)	(13, 34)	
HVLT Delayed Recall				
Median (IQR)	9 (3)	9 (3)	8 (4)	0.09
(Min, max)	(0, 12)	(0, 12)	(0, 12)	
HVLT Delayed Recognition				
Median (IQR)	12 (1)	12 (1)	12 (1)	0.733
(Min, max)	(0, 12)	(0, 12)	(6, 12)	
HVLT Retention				
Median (IQR)	0.9 (0.25)	0.9 (0.25)	0.89 (0.27)	0.217
(Min, max)	(0, 1.29)	(0, 1.29)	(0, 1.22)	
Judgment of Line Orientation				
Median (IQR)	13 (2.5)	13 (2.5)	13 (2.5)	0.9464
(Min, Max)	5, 15	5, 15	6, 15	
Semantic Fluency test				
Mean (SD)	48.62 (11.64)	49.2 (11.36)	46.5 (11.9)	0.1007
(Min, max)	20, 103	24, 91	20, 103	
Letter Number Sequencing				
Mean (SD)	10.58 (2.66)	10.55 (2.67)	10.63 (2.63)	0.7706
(Min, max)	2, 20	2, 20	4, 17	
MCI (no, %)				
Normal cognition	346 (84.3)	253 (60.24%)	93 (22.14%)	0.7025
MCI	65 (15.7)	46 (10.95%)	19 (4.5%)	
Geriatric Depression Scale				
Median (IQR)	2 (2)	2 (2)	2 (2)	0.545
(Min, max)	(0, 14)	(0, 13)	(0, 14)	

HVLT, Hopkins Verbal Learning Test; IQR, interquartile range; MCI, mild cognitive impairment based on >1.5 SD below mean on two cognitive test scores; MDS‐UPDRS, Movement Disorder Society‐Sponsored Revision of the Unified Parkinson's Disease Rating Scale; MoCA, Montreal Cognitive Assessment; SD, standard deviation.

^a^ Data derived from the Parkinson Progression Marker Initiative (PPMI).^7^

*** *p*‐Value <0.05.

### Association between NLR and cognitive performance

**Figure 2:**
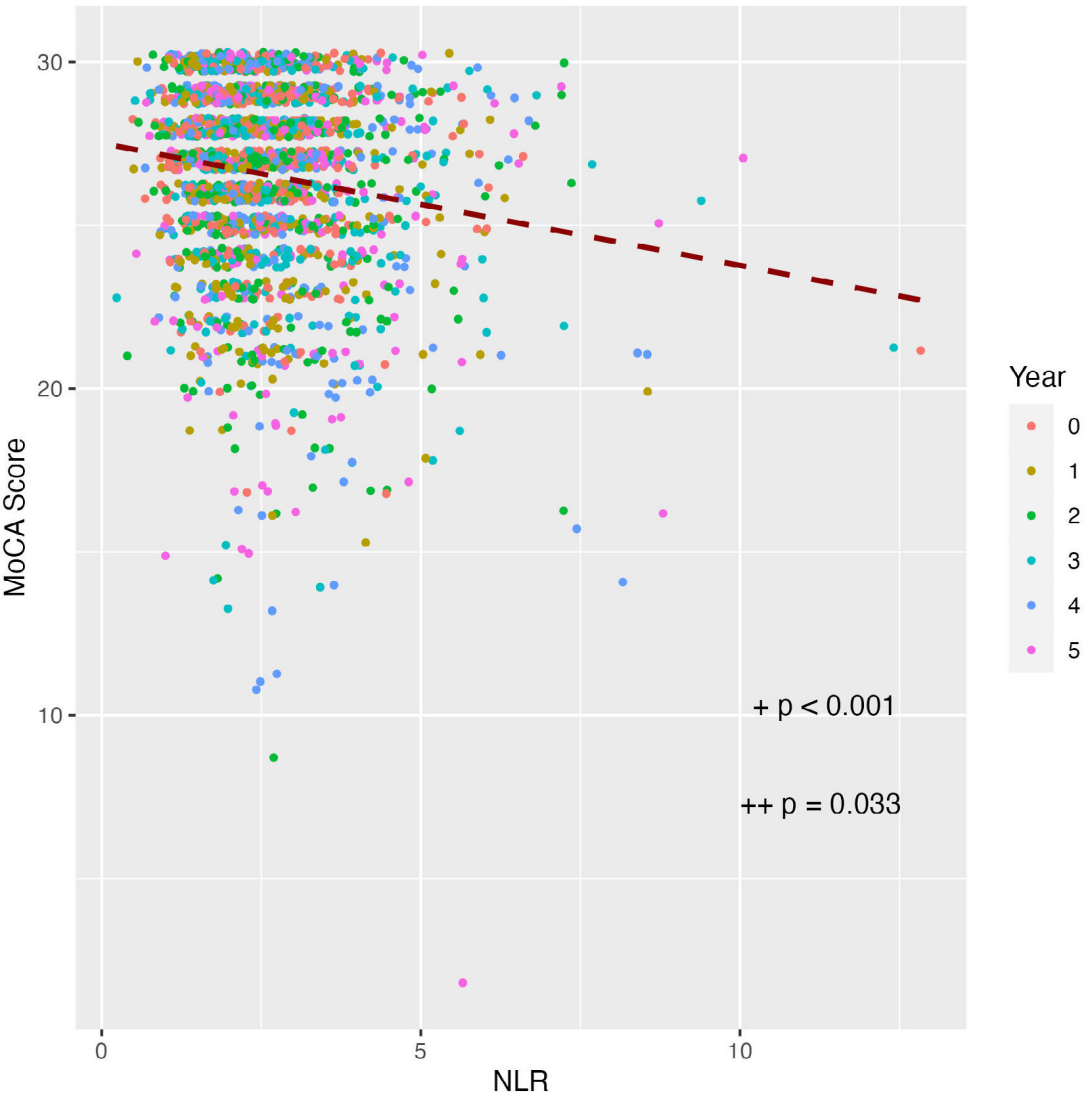
Association between neutrophil‐to‐lymphocyte ratio (NLR) and Montreal Cognitive Assessment (MoCA) score. ^+^ *p*‐value from the unadjusted model. ^++^ *p*‐value of the model after controlling for covariates.

There was a statistically significant negative association between NLR and MoCA scores (*b* = −0.20 *p* < 0.001); for each one‐unit increase in NLR, we observed a decrease of approximately 0.2 points in the MoCA score, after accounting for patient‐specific random effects in the primary model (Fig. 2).

After adding relevant covariates to this model, the negative association was still significant (*b* = −0.12 *p* = 0.033), adjusted for age, sex, education, APOE genotype, MDS‐UPDRS Part III score, RBD, and duration of PD. Each incremental one‐unit increase in NLR was associated with a decrease of approximately 0.1 points in the MoCA score. Detailed findings from the association between NLR and MoCA from the linear mixed‐effects model, with adjustments for covariates, are provided in Table 3.

**Table 3:** Linear mixed‐effects model of association between NLR and MoCA.

Variable	Estimate	SE	*t*‐Value	*p*‐Value
MoCA	−0.206	0.0546	−3.771	<0.001***
Age	−0.078	0.0116	−6.67	<0.001***
Female sex	0.0813	0.2362	3.442	<0.001***
Education years	0.1243	0.0387	3.218	0.001***
MDS‐UPDRS Part 3	−0.0308	0.0056	−5.503	<0.001***
RBD	−0.0121	0.0247	−0.493	0.6221
APOE genotype				
e2/e2	−0.6648	1.641	−0.395	0.6930
e2/e4	−0.1519	0.987	−0.154	0.8777
e3/e2	0.5031	0.4970	0.999	0.3183
e3/e3	0.4855	0.4056	1.197	0.232
e4/e3	0.0986	0.4521	0.218	0.8275
e4/e4	−0.2909	0.8445	−0.345	0.7306
Duration of PD	0.0060	0.0173	0.0418	0.7279
MoCA (adjusted for covariates)	−0.1241	0.0581	−2.135	0.0329***

APOE, apolipoprotein E genotype; MDS‐UPDRS, Movement Disorder Society‐Sponsored Revision of the Unified Parkinson's Disease Rating Scale; NLR, neutrophil‐to‐lymphocyte ratio; RBD, REM sleep behavior disorder; SDMT, Symbol Digit Modalities Test; SE, standard error.

*** *p*‐Value <0.05.

Our investigation of covariates revealed some associations with global cognitive performance, as measured by MoCA (Table 3). As expected, older age (*b* = −0.08, *p* < 0.0001), male sex (*b* = 0.08, *p* < 0.001), fewer years of education (*b* = 0.12, *p* = 0.001), and greater MDS‐UPDRS Part III scores (*b* = −0.03, *p* < 0.0001) were associated with worse MoCA scores. APOE genotype, presence of RBD, and duration of PD were not associated with MoCA scores.

**Figure 3:**
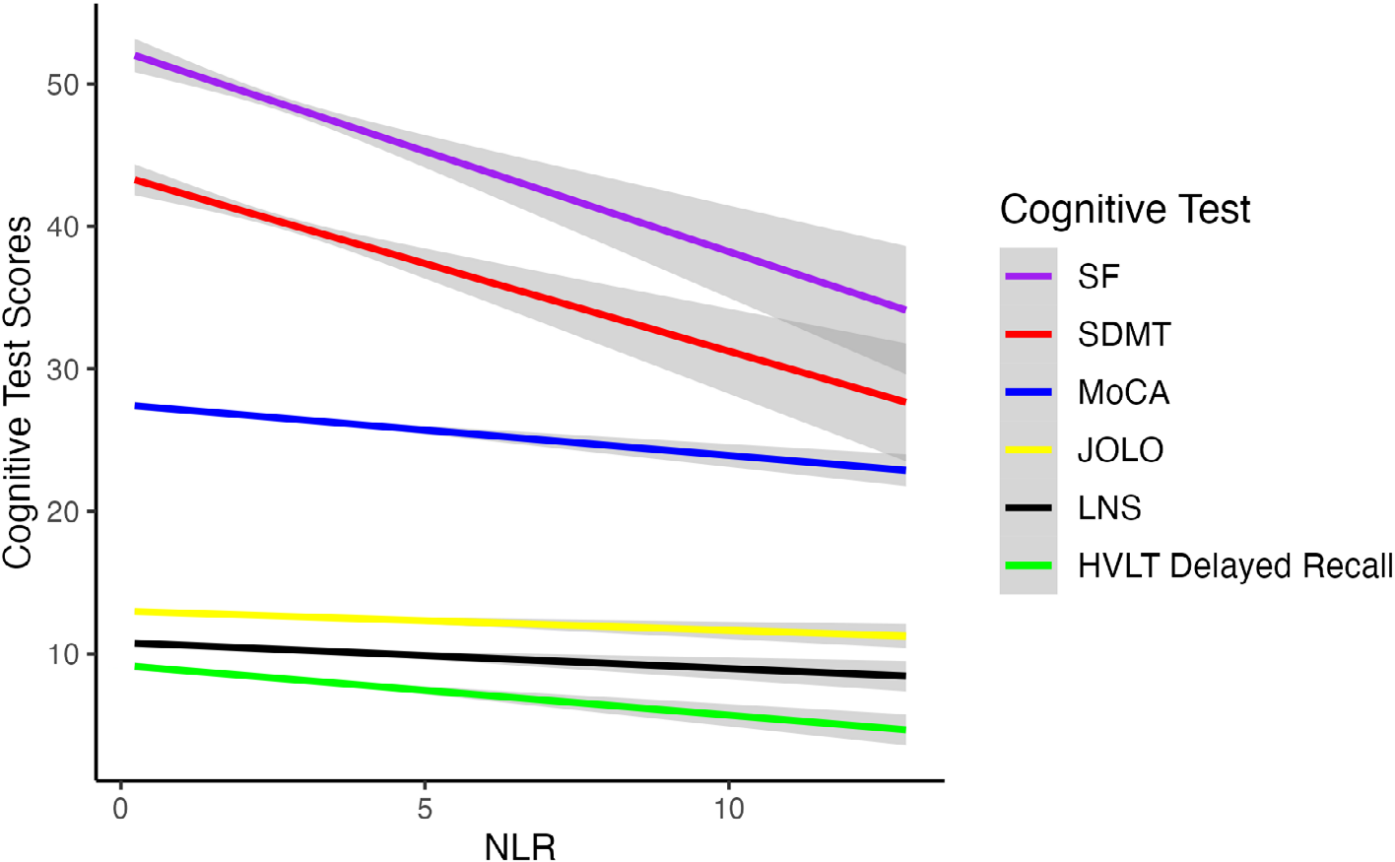
Association between neutrophil‐to‐lymphocyte ratio (NLR) and Symbol Digit Modalities Test (SDMT) score. ^+^ *p*‐value from the unadjusted model. ^++^ *p*‐value of the model after controlling for covariates.

We examined associations of NLR with other tests of cognitive performance, such as the SDMT, which contains sensitive measures of processing speed, attention, and working memory. For the SDMT, there was a highly statistically significant, negative association between NLR and SDMT total score (*b* = −0.84, *p* < 0.0001, Fig. 3). We then added covariates to this model, and after controlling for age, sex, education, MDS‐UPDRS Part III score, APOE genotype, RBD, and duration of PD, the association between NLR and SDMT performance was still present (*b* = −0.61, *p* < 0.001, Table 4).

**Table 4:** Association between NLR as a predictor variable and cognitive performance without time interaction (results of adjusted models shown).

Variable	Estimate	SE	*t*‐Value	*p*‐Value
SDMT	−0.6123	0.1808	−3.386	0.0007***
HVLT Delayed Recall	−0.1777	0.0564	−3.151	0.0017***
Judgment of Line Orientation	−0.1227	0.0456	−2.691	0.0072***
Semantic Fluency test	−0.088	0.1966	−0.447	0.654
Letter Number Sequencing	−0.1143	0.0519	−2.2	0.028***

Separate individual models were generated each to model SDMT, HVLT Delayed recall, JOLO, SF, and LNS (adjusted for age, sex, years of education, APOE status, MDS‐UPDRS III, Duration of PD, and presence of RBD).

HVLT, Hopkins Verbal Learning Test; MoCA, Montreal Cognitive Assessment; NLR, neutrophil‐to‐lymphocyte ratio; SDMT, Symbol Digit Modalities Test; SE, standard error.

*** *p*‐Value <0.05.

**Figure 4:**
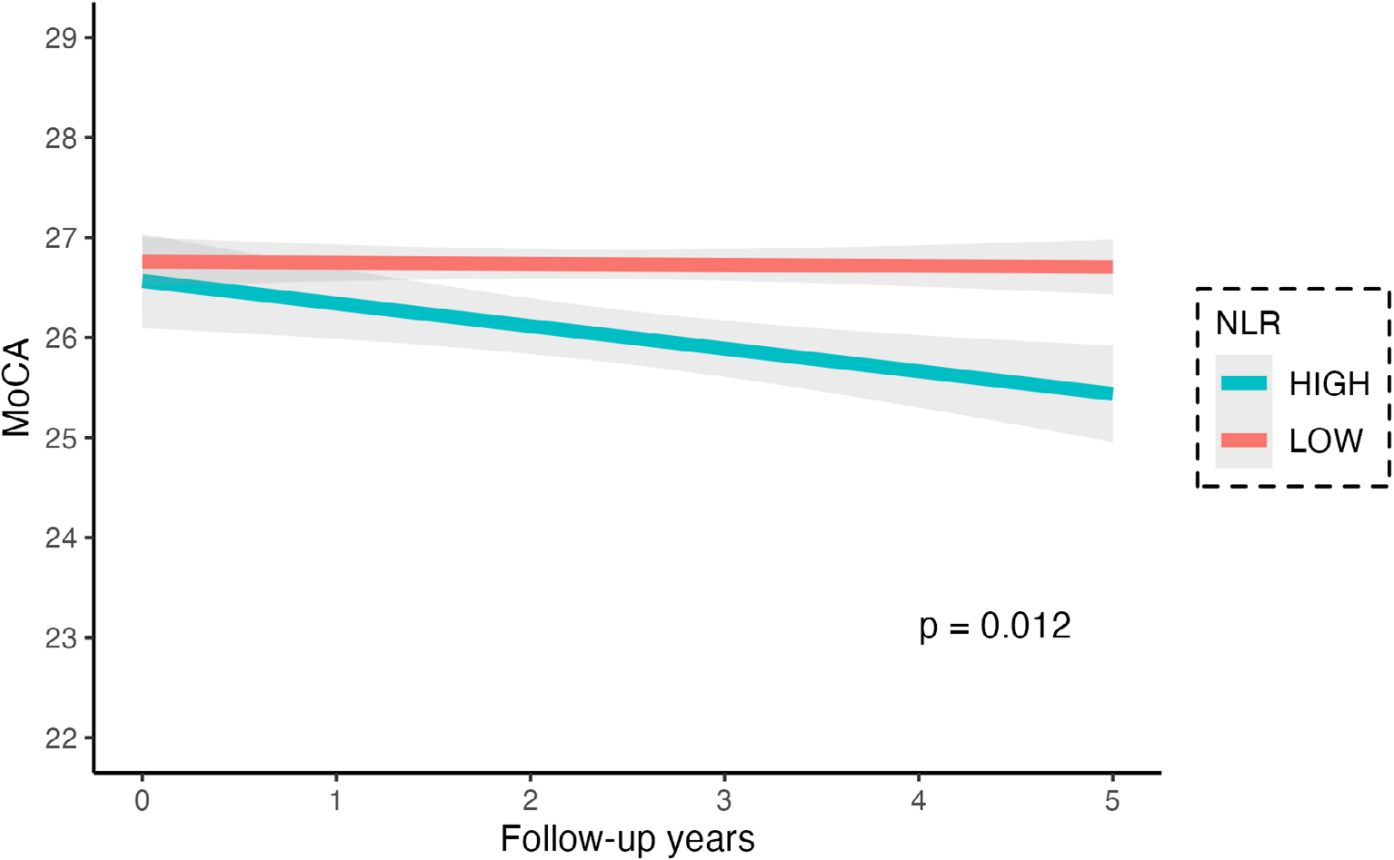
Association between neutrophil‐to‐lymphocyte ratio (NLR) and six cognitive tests scores.

Similarly, we examined the association between NLR and HVLT Delayed Recall score. There was a statistically significant negative association (*b* = −0.23, *p* < 0.001) in the unadjusted model, and in the adjusted model using the same covariates (*b* = −0.18, *p* = 0.002, Table 4). In additional adjusted analyses, there was a statistically significant negative association between NLR and JOLO (*b* = −0.12, *p*‐value <0.01) and LNS (*b* = −0.11, *p* = 0.027). However, we found no significant association between NLR and total SF in either the unadjusted (*b* = −0.28, *p* = 0.11) or the adjusted model (*b* = −0.09, *p* = 0.656, Fig 4, Table 4).

**Figure 5:**
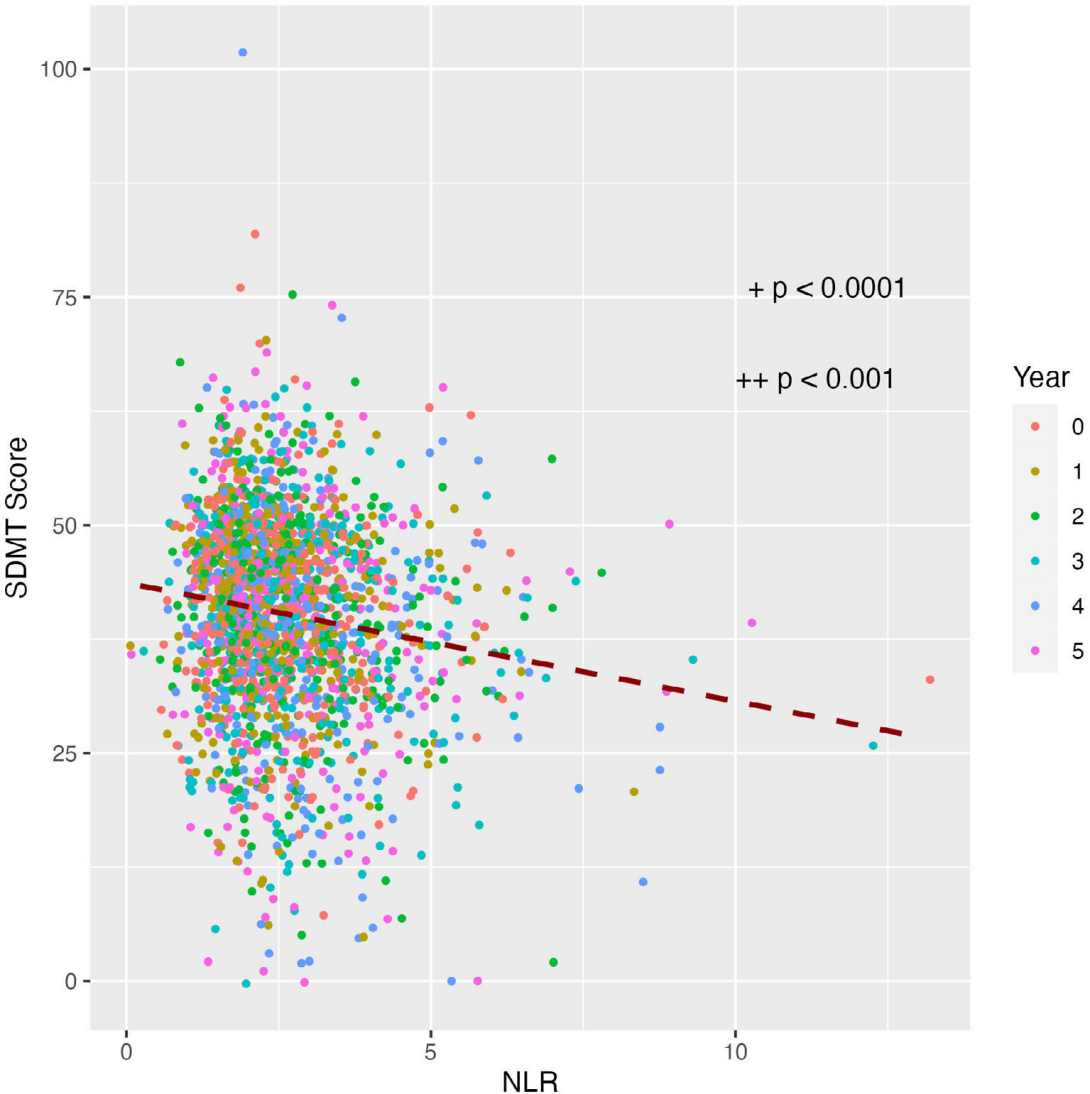
MoCA score over 5 years of follow‐up in Parkinson's disease (PD) participants with high neutrophil‐to‐lymphocyte ratio (NLR) (NLR >3) and low NLR (NLR ≤3).

Next, to study whether NLR influenced change in cognitive performance over time, we introduced an interaction term (year) into the model to investigate the temporal influence on the association between NLR and each of the cognitive tests over time (Table 5). After adjusting for covariates, there remained a statistically significant time interaction (*b* = −0.07, *p* = 0.005), with greater NLR exhibiting a significant decline in MoCA over time (Fig. 5, Table 5).

**Table 5:** Linear mixed‐effects models with time interaction for NLR and MoCA, SDMT, HVLT Delayed Recall, JOLO, SFT, and LNS (results of adjusted models shown).

Variable	Estimate	SE	*t*‐Value	*p*‐Value
MoCA				
NLR:year	−0.0655	0.0233	−2.813	0.005 ***
SDMT				
NLR:year	−0.099	0.0700	−1.412	0.1581
HVLT Delayed Recall				
NLR:year	−0.0244	0.0227	−1.071	0.2843
JOLO				
NLR:year	0.0114	0.0186	0.614	0.5392
Semantic Fluency test				
NLR:year	−0.1101	0.0747	−1.475	0.140
Letter Number Sequencing				
NLR:year	−0.0354	0.0202	−1.749	0.0806

HVLT, Hopkins Verbal Learning Test; JOLO, Benton Judgment of Line Orientation Test; LNS, Letter Number Sequencing; MoCA, Montreal Cognitive Assessment; NLR, neutrophil‐to‐lymphocyte ratio; SDMT, Symbol Digit Modalities Test; SE, standard error; SFT, Semantic Fluency Test; year, time interaction (year).

*** *p*‐Value <0.05.

### NLR categories and cognitive performance

To determine whether more clinically useful NLR categories would have similar significant associations with cognitive performance, we applied linear mixed‐effects models to each of the cognitive tests, with and without a time interaction. The unadjusted model showed a significant association between the high NLR group and MoCA (*b* = −0.27, *p* = 0.038), suggesting that participants in this group had MoCA scores that were on average 0.3 points lower than those in the low NLR group. However, after adjusting for age, sex, education years, APOE genotype, MDS‐UPDRS part III score, and RBD, the association between an NLR >3 and MoCA score was not statistically significant (*b* = −0.17, *p* = 0.23, Table S1).

Similarly, when analyzing associations with SDMT, there was a significant negative association with high NLR (*b* = −1.67, *p* < 0.0001), and after controlling for age, sex, education, MDS‐UPDRS III, APOE genotype, and presence of RBD, this remained statistically significant (*b* = −1.16, *p* = 0.008, Table S1). We did observe a significant negative association between high NLR and LNS scores (*b* = −0.28, *p* = 0.013), which persisted even after adjusting for covariates (*b* = −0.26, *p* = 0.04). Similar unadjusted analyses showed an association between high NLR and HVLT Delayed Recall (*b* = −0.34, *p* = 0.005), but after controlling for covariates, this association was not statistically significant (*b* = −0.25, *p* = 0.08). Finally, analyses between high NLR and JOLO performance and between high NLR and SF performance were not significant.

We also analyzed the association between NLR categories and cognitive performance for each test over time. To study whether NLR influenced change in cognitive performance over time, we introduced an interaction term (year) into the model to investigate the temporal influence on the association between cognitive performance scores and NLR groups (Table 5). After adjusting for covariates with the time interaction, high NLR was associated with MoCA (*b* = −0.07, *p* = 0.005, Table S1) and with LNS (*b* = −0.15, *p* = 0.006), but for each of the other cognitive tests, the interaction with time was not significant.

### Association between NLR and MCI status

Finally, we examined NLR association with MCI status over 5 years of follow‐up, as defined by MDS PD‐MCI Level 1 criteria (at least two cognitive test scores >1.5 standard deviations below standardized mean). Excluding those with MCI status at baseline, there were 307 participants whose MCI status was available over 5 years of follow‐up, with 56 reported as having MCI at the end of the 5‐year follow‐up. After excluding PD participants with MCI at baseline, using a mixed‐effects logistic regression model with time interaction, there was no statistically significant association between NLR as a continuous variable and MCI over 5‐year follow‐up (*b* = 0.03, *p* = 0.65). Similarly, no significant relationship was observed between the presence of a high NLR at baseline and the likelihood of developing MCI over the 5‐year follow‐up period (*b* = 0.07, *p* = 0.65).

## Discussion

Our findings suggest significant associations between NLR and abbreviated measures of global cognitive performance, as measured by MoCA. This association remained significant in predicting MoCA over time, even after controlling for relevant covariates such as age, sex, education, PD motor severity, PD duration, APOE genotype, and presence of RBD. Additionally, we observed significant associations between NLR and other measures of cognition, namely those like the SDMT, which is sensitive to detecting deficits in cognitive performance in the domains of processing speed, attention and working memory. Cognitive performance in domains such as verbal memory (HVLT‐Delayed Recall), visuospatial function (JOLO), and working memory (LNS) were also associated with NLR, such that the greater the NLR, the worse performance in these cognitive domains. Collectively, these data suggest a discernible link between NLR and cognitive performance.

However, there was no link between NLR and change in cognitive performance over time on tests other than MoCA. We also found no trend towards impairment on amnestic (e.g., verbal memory) versus non‐amnestic (e.g., visuospatial or working memory) patterns of cognitive performance, contrary to prior associations reported in the literature between NLR and incident AD, and a recent case–control study showing that NLR correlated inversely with AD biomarkers, namely, CSF amyloid‐β42.^51^

Although there is frequent AD co‐pathology of amyloid and tau in individuals with PD, both in‐life^52^ and postmortem,^53^ and it is known that individuals with PD are at risk for developing AD‐type cognitive changes, our analyses revealed no discernible link between APOE genotypes and MoCA scores. Furthermore, when exploring the relationship between these genotypes and the other cognitive tests, our findings also did not support an expected negative impact of the ε4 allele or a protective effect often suggested for the ε2 allele, as previously reported in the literature.^20^, ^52^

We also did not see statistically significant associations between clinically interpretable categories of high NLR (NLR >3) or low NLR (NLR ≤3) groups with cognitive performance in the mixed‐effects model. There may be several reasons for this, including information loss during categorization. Therefore, while categorization might help in simplifying the interpretation and potentially improve its applicability in the clinic, it may obscure more complex relationships present in the data.

Baseline NLR levels did not predict the development of MCI over the 5‐year follow‐up period among participants who did not initially have MCI. MCI status, NLR values, and their interaction may change over time in individuals with PD. NLR measured throughout the study may be better than baseline NLR characterization to capture the association between cognition and peripheral inflammatory status for an individual. In other words, NLR may not be as much a predictor of conversion to MCI as much as an indicator of contemporaneous cognitive performance. However, our ability to test the association between NLR and incident dementia was limited as there are few reported individuals with dementia in the PPMI cohort. Future studies using other cohorts that employ ongoing dementia surveillance might be used to explore the relationship between NLR and progression to dementia.

There were several strengths to this study. This study employs a longitudinal design, with standardized validated cognitive assessments allowing for the examination of cognitive change over time. It also includes a substantial number of participants, enhancing the statistical power and generalizability of the findings. Access to an extensive array of covariate data from the PPMI database provided a rich source for analysis. Additionally, by incorporating these potential covariates in the statistical models, the study systematically controlled for their effects. This approach enhances the accuracy of the results, enabling a comprehensive view of the association between NLR and cognitive impairment. The use of mixed‐effects models accounts for the inherent variability within and between subjects, providing a robust statistical approach for analyzing repeated measures data.

While the available data allowed for a substantial sample size to derive robust findings, it is important to acknowledge that there were limitations to our study. There was incomplete data, primarily attributable to some patients not completing all scheduled visits during the follow‐up period. To effectively address this, we used linear mixed‐effects models, enabling us to leverage the entirety of the available data by accommodating the inherent variability in visit attendance. Furthermore, the PPMI study intentionally excluded people with dementia at baseline and recruited a highly educated and cognitively intact sample of people with PD.

This study highlights the potential utility of monitoring NLR levels as an indicator of day‐to‐day cognitive performance, in a highly selected, cognitively healthy cohort. The lack of follow‐up to dementia may preclude us from drawing conclusions about whether NLR is important for predicting progression in cognitive impairment status to dementia over time. Our findings indicate a negative association between NLR and cognitive performance in *de novo* PD patients over a 5‐year follow‐up period. However, future studies including patients with more diverse socioeconomic, educational backgrounds and longer term cognitive trajectories could yield results that are more broadly applicable to a wider population. Our findings add to the current knowledge about the effects of peripheral inflammation in PD with respect to cognitive performance in this population.

## Author Contributions

J.L: Conceptualized the study, designed the methodology, conducted data collection and analysis, drafted the manuscript, prepared tabes and figures. L.S: Supervise the project, contributed to the study design and methodology, drafted the manuscript, and provided continuous feedback. J.W: Contributed to study design and statistical methods. Reviewed the manuscript and provided critical feedback. A.G: Reviewed and edited the manuscript for important intellectual content. Contributed to the final manuscript. All authors read and approved the final version of the manuscript.

## Conflict of Interest

The authors declare that they have no conflicts of interest regarding this research study.

## Funding Information

This research received no external funding.

## Supporting information


Table S1.

## Data Availability

The data that support the findings of this study are available from the corresponding author upon reasonable request.
