# Supplementary material for: Neutrophil‐to‐lymphocyte ratio and longitudinal cognitive performance in Parkinson's disease
Source: Ann Clin Transl Neurol. 2024 Jul 19;11(9):2301–13. doi: 10.1002/acn3.52144 (PMC11537143; doi:10.1002/acn3.52144)
Supplement: Supplementary file 1 — Table S1. [file ACN3-11-2301-s001.docx]

| Supplementary Table 1. Adjusted Linear Mixed Effects Models for High NLR Category with and without Time Interaction | | | | |
| --- | --- | --- | --- | --- |
| **Variable** | **Estimate** | **SE** | **t-value** | **p-value** |
| **MoCA** |  |  |  |  |
| High NLR | - 0.1707 | 0.1413 | - 1.208 | 0.227 |
| High NLR:Year ^+^ | - 0.1620 | 0.0645 | - 2.513 | 0.012 *** |
| **SDMT** |  |  |  |  |
| High NLR | - 1.1605 | 0.4360 | - 2.659 | 0.008 *** |
| High NLR:Year ^+^ | - 0.2507 | 0.1954 | - 1.283 | 0.2 |
| **HVLT Delayed Recall** | | | | |
| High NLR | -0.2405 | 0.1378 | -1.745 | 0.081 |
| High NLR:Year ^+^ | -0.0814 | 0.0631 | -1.289 | 0.198 |
| **JOLO** | | | | |
| High NLR | -0.1306 | 0.1114 | -1.173 | 0.241 |
| High NLR:Year ^+^ | -0.0221 | 0.0513 | -0.431 | 0.667 |
| **SF Total** | | | | |
| High NLR | -0.4162 | 0.4713 | -0.883 | 0.377 |
| High NLR:Year ^+^ | -0.2976 | 0.2084 | -1.428 | 0.153 |
| **Letter Number Sequencing** | | | | |
| High NLR | -0.2578 | 0.1256 | -2.053 | 0.040 *** |
| High NLR:Year ^+^ | -0.1547 | 0.0562 | -2.755 | 0.006 *** |
| Abbreviations: SE, Standard Error; MoCA, Montreal Cognitive Assessment, SDMT, Symbol Digit Modalities Test; HVLT, Hopkins Verbal Learning Test; JOLO, Benton Judgment of Line Orientation Test; NLR, Neutrophil-to-Lymphocyte Ratio; *** p-value <0.05 | | | | |
